# Efficacy comparison of Kirschner-wire tension band combined with patellar cerclage and anchor-loop plate in treatment of inferior patellar pole fracture

**DOI:** 10.3389/fbioe.2022.1010508

**Published:** 2022-10-17

**Authors:** Bing Du, Teng Ma, Huanan Bai, Yao Lu, Yibo Xu, Yanling Yang, Kun Zhang, Zhong Li, Ming Li

**Affiliations:** ^1^ Honghui Hospital, Xi’an Jiaotong University, Xi’an, China; ^2^ Medical College of Yan’an University, Yan’an, China

**Keywords:** patellar fractures, tension band, finite element analysis, plates, inferior pole

## Abstract

**Objective:** This study aimed to compare the biomechanical stability and clinical efficacy of the Kirschner-wire (K-wire) tension band combined with patellar cerclage and an anchor-loop plate (ALP) in treating inferior-pole patellar fracture.

**Methods:** The finite element model was established to analyze the mechanical properties of a K-wire tension band combined with patellar cerclage and ALP fixation in the treatment of inferior patellar pole fracture. The clinical data of 49 patients with patellar inferior-pole fracture (AO/OTA 34 A1) admitted to our hospital from January 2017 to July 2021 were retrospectively analyzed. Among these, 28 cases were fixed with ALPs (ALP group) and 21 cases were fixed with K-wire tension bands combined with patellar cerclage (K-wire group). By reviewing the medical records and follow-up results, we compared the operation time, final knee joint activity, incidence of secondary surgery, postoperative complications, and joint function recovery between the two groups.

**Results:** The biomechanical analysis of the finite element model showed that the maximum displacement of the K-wire group was 1.87 times that of the ALP group. The maximum stress of the K-wire group was 1.34 times that of the ALP group. The maximum stress of the pole bone in the K-wire group was 13.89 times that of the ALP group. The average follow-up times of the K-wire group and ALP group were similar (*p* > 0.05), and the average ages of the two groups were similar (*p* > 0.05). The operation time of the ALP group was significantly shorter than that of the K-wire group (*p* < 0.05).The final knee joint activity of the ALP group was significantly greater than that of the K-wire group (*p* < 0.05). The Bostman patellar fracture function score of the ALP group was significantly better than that of the K-wire group at 3 and 9 months after operation (*p* < 0.05). Postoperative complications of the two groups included 1 case (3.6%) in the ALP group with internal fixation-stimulation complications and, in the K-wire group, 3 cases (14.3%) with internal fixation stimulation complications and 1 case (4.8%) with infection.

**Conclusion:** The ALP and K-wire tension band combined with patella cerclage models were tested at 500 N, and no damage occurred, indicating that the newly designed ALP is safe in mechanical structure. The ALP has better therapeutic effect in biomechanical stability, postoperative complications, secondary surgery, and knee function. This technique is an effective method for the treatment of inferior-pole patellar fracture.

## Background

The patella is the most prominent seed bone in the human body and a part of the knee joint. It plays a vital role in transmitting quadriceps femoris strength and in the composition of knee extension devices (Zhang et al., 2020). The knee extension device is a complete knee extension structure composed of the quadriceps femoris and tendons, patella, patellar ligament, and tibial nodules (Brooks, 2009). A large-scale epidemiological survey found that patellar fracture accounted for 77.5% of knee extension device injuries, followed by patellar ligament rupture (13.5%) and quadriceps femoris rupture (9%) (Lyons et al., 2022). It is clear that patellar fracture is the primary cause of injury to a knee extension device. We believe that restoring the continuity of the knee extension device should be the focus of treating the patellar fracture. Among the patellar fractures, the incidence of inferior-pole fracture accounts for 9.3%–22.4% (Matejcić et al., 2008). Fracture of the inferior patella usually refers to a fracture of the distal one-fourth of the patella (Gwinner et al., 2016). The fracture block of the inferior pole of the patella is usually tiny and crushed, and it is difficult to fix and maintain anatomical reduction (Oh et al., 2015), which poses a challenge for the treatment of this fracture. The inferior patellar pole is connected to the patellar ligament.

The tension band technique has been widely used in the treatment of patellar fractures (Kakazu and Archdeacon, 2016), and it is reported that this technique is applied to treat patellar inferior-pole fractures (Yang et al., 2017; Lu et al., 2021). However, when applied to the inferior-pole fracture of the patella, it may easily lead to the failure of internal fixation (Zhu et al., 2020). Partial resection of the patella and reconstruction of the patellar ligament may affect the patella’s short and long axes and affect the patellofemoral joint’s function in the treatment of inferior-pole fracture of the patella (Huang et al., 2021). Comparatively better outcomes have been achieved by the fixation of displaced fragments (Veselko and Kastelec, 2005; Matejcić et al., 2008).

There are many surgical options for the treatment of patellar inferior-pole fracture, such as the Kirschner-wire (K-wire) tension band technique, cannulated lag screws (Chang and Ji, 2011), tension band wiring (Yang et al., 2017), separate vertical wiring (Song et al., 2014; He et al., 2018), and basket plates (Veselko and Kastelec, 2005). The K-wire tension band technique is one of the most used methods of treating patellar fracture. However, due to its complications, it often requires reoperation or revision to remove internal fixation. At present, the method to obtain a certain therapeutic effect is a tension band combined with patellar surface cerclage (Wang et al., 2021). However, there is still no recognized surgical treatment. To solve the treatment problem of patellar inferior-pole fracture, we designed a new surgical method, namely, anchor-loop plate (ALP) fixation, which combines microplate and wire ring fixation. As with the traditional K-wire tension band in the treatment of patellar lower-pole fracture, but unlike the traditional fixation method, the patella inferior pole and patellar ligament are joined together by the plate in the suprapatellar pole bed, and then steel-wire ring ligation makes joins them into a whole, realizing the immediate recovery of the knee extension device. Patients can then perform early functional exercises, dramatically reducing tension near the fracture line, to avoid stress concentration near the fracture line caused by bone nonunion, reduction loss, and other postoperative complications. This study used a combination of clinical research and finite element model biomechanical analysis to compare the biomechanical characteristics of the ALP and tension band combined with patellar cerclage in the fixation of the inferior patellar fracture.

## Materials and methods

### Finite element analysis: Collection of imaging data

A 35-year-old male weighing 70 kg was selected, as a patient with a patellar fracture and who voluntarily signed the informed consent. X-ray examination showed that the healthy side of the patient’s patella had no disease and good bone. A spiral CT scan was used; the scanning target was the healthy side of the patient’s patella. The scanning length was from 5 cm above the superior pole of the patella to 5 cm below the inferior pole of the patella. The scanning conditions were 120 kV and 155 mA, and the scanning layer thickness was 1 mm. The scanning information was collected and processed, and the continuous image data were saved in DICOM format.

### Finite element model of patella construction

The data was imported into Mimics 15.0 software, and the rough femur model was established by threshold segmentation, regional growth, and other software commands. The model was imported into Geomagic 2017 software and subdivided into triangular facets, noise reduction, and smooth processing. The three-dimensional model of the patella’s cancellous and cortical bone was constructed by accurate surface processing. This model was imported into Solidworks software to simulate the entire reconstruction of the inferior-pole fracture of the patella, and the fracture line was cut within a quarter of the patella. The experiment was divided into two groups: A) a K-wire group, in which K-wires parallel to the patella’s articular surface penetrate the patella and are bundled with an 8′steel-wire cerclage of the patella surface, and B) an ALP group in which the microplate is bent and attached to the surface of the patella through the patellar ligament, fixed with three screws and one wire.

### Volume mesh generation

**FIGURE 1:**
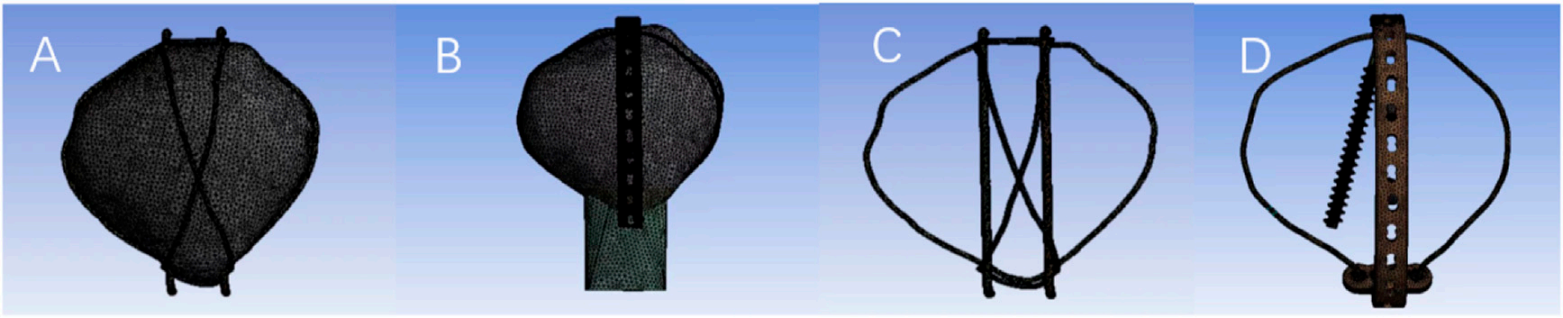
Model after meshing.

Finite element models were constructed using linear tetrahedrons (Figure 1). This study showed 414956 elements with 552436 nodes in the tension band model after meshing and 238455 elements with 309992 nodes in the microplate model. The specific values are shown in Table 1.

**TABLE 1:** Number of nodes and elements in finite element model.

Number of nodes and elements in finite element model		
Finite element model	Nodes	Elements
A group (K-wire)	552436	414956
B group (ALP)	309992	238455

### Assignment of material properties

The related material properties of a calcaneal fracture finite element model were set up to establish a 3D finite element model similar to the practical model in terms of its material parameters and mechanical behavior. The ligaments were set up as linear elastic materials sustaining tensile stress only. According to the actual data of clinical internal-fixation devices, the two kinds of internal-fixation structures involved in this study were constructed by Solidworks software, and the material properties of patellar cortical bone, patellar cancellous bone, K-wire, steel wire, and titanium alloy (plate and screw) were set up (Meng et al., 2017; Ling et al., 2019; Kim et al., 2021). The specific parameters are shown in Table 2. The 3D model of the patella and the internal-fixation model were assembled in SolidWorks software.

**TABLE 2:** Model material parameters.

Name of the material	Elastic modulus (MPa)	Poisson ratio
Cortical bone	10000	0.30
Cancellous bone	840	0.29
Steel wire	100000	0.29
Kirschner wire	200000	0.30
Titanium alloy	110000	0.30

### Constraint and loading

Two groups of three-dimensional finite element models were fixed in the patellar fracture model. Forces of 0–500 N (He et al., 2018) were applied on the surface of patella inferior pole within 1 s.

### Clinical data

From January 2017 to July 2021, 49 patients with patellar fracture (AO/OTA 34 A1) were included in this study. Patients were divided by different fracture fixation methods into the K-wire tension band–patellar cerclage group or the ALP group. The hospital ethics committee approved the experiment, and all patients signed the informed consent. The inclusion criteria were as follows: 1) CT or X-ray diagnosis of inferior pole patellar fracture; 2) acceptance of K-wire tension band combined with patellar cerclage fixation and anchor ring fixation; 3) the completion of the operation within one week of injury; and 4) the obtaining of patients’ informed consent and complete clinical data. Patients were excluded based on the following criteria: 1) other types of patellar fractures; 2) multiple fractures; 3) combined patella, upper pole fracture, or patellar ligament injuries; 4) severe osteoarthritis or peri-knee surgery history; and 5) loss of follow-up or less than 9 months of follow-up.

### Surgical procedure

#### Anchor-loop plate group

**FIGURE 2:**
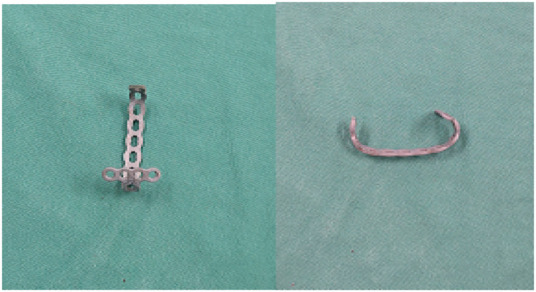
Microplate formed by bending.

The main body of the ALP used in this operation is made of microplate bending (Figure 2; Tianjin Zhengtian Company).

**FIGURE 3:**
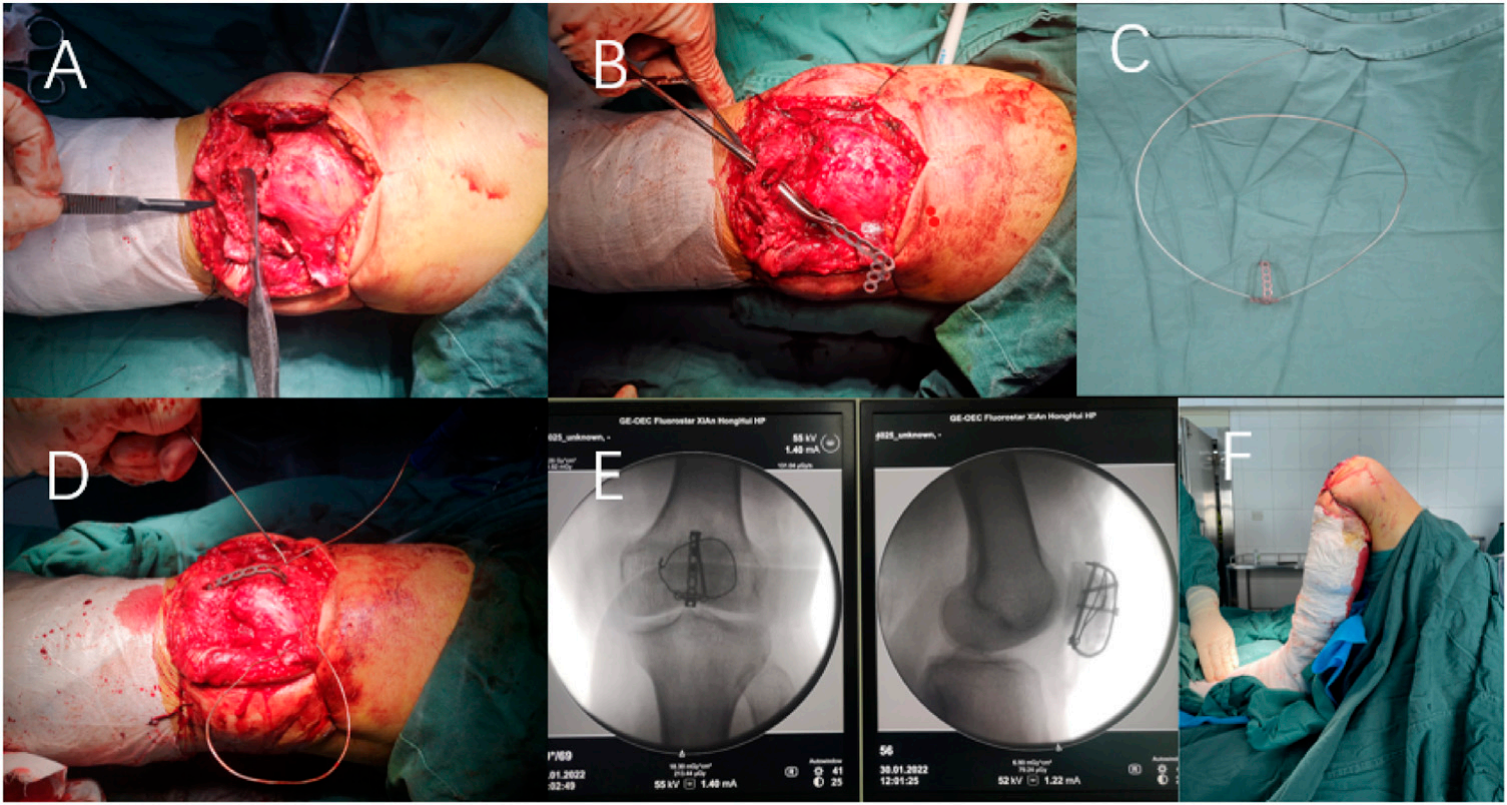
Intraoperative situation of ALP in the treatment of inferior pole patellar fracture. **(A)** Incision of a small opening at the junction of the patellar ligament and inferior pole with a scalpel. **(B)** One end of the long arm of the pre-flexed plate passes through the small opening of the patellar ligament. **(C)** Plasticized bone plate penetrated steel wire. **(D)** Ring wire assisted by lumbar puncture after fracture reduction. **(E)** Intraoperative fluoroscopy. **(F)** Immediate joint passive flexion and extension activity.

After the patient was satisfied with anesthesia, they took a supine position while routine disinfection was performed, the sterile surgical scarf spread, and the tourniquet placed near the thigh. In the operation, an anterior longitudinal median incision was used to expose the fracture, flush the articular cavity, clean the blood stasis and clot around the joint and the fracture end, and confirm the fracture type. A knife was used to cut a small opening on the surface of the connection between the patellar ligament and the lower pole, and the long arm of the steel plate was passed through the patellar ligament from the inside to the outside. Then, the short arm of the steel plate was dragged along the lower pole of the patella, and the steel plate was adjusted with the assistance of a perspective machine and attached to the surface of the patella. After satisfactory shaping, the steel wire was penetrated. Once again, the long arm of the steel plate was passed through the small incision of the patellar ligament from the inside to the outside, and the lower pole was dragged to reset. In cases of severe crushing, sutures were used to close the lower bone fragments and suture them to the short arm hole of the anchor plate. Steel wire puncture was assisted by lumbar puncture needle, leading to penetration of the medial and lateral patellar retinaculum and soft tissue, and the steel wire loop was tightened (the diameter of the steel wire was 1 mm). Then the screw (all 2.7 system screws) was used to fix the bone plate. During the operation, the knee joint was buckled, and the stability of the fracture was examined. Large amounts of physiological saline were repeatedly used to rinse the wound. The patellar ligament incision was sutured, the aligned flap was sutured, and the skin was sutured layer by layer. The knee joint’s passive flexion and extension activity was observed and recorded immediately. The key intraoperative steps are shown in Figure 3.

#### K-wire group

The methods of anesthesia and fracture exposure were the same as in the ALP group. The internal-fixation operation was performed according to the standard of the K-wire tension band for the fracture of the lower pole of the patella, then wire cerclage on the surface of the patella further secured the fixation.

### Outcome measures

On the second day after the operation, the dressing was opened to observe the wound, and the wound drainage tube was removed. The wound was strictly disinfected and the dressing replaced. X-ray and CT scans of the knee joint were retrospectively analyzed. At the beginning of knee flexion and extension exercises, the movement angle gradually increased (the movement target angle was 90° one week after the operation). Half-weight-bearing exercise began 1 day after the operation, full-weight-bearing training was performed beginning 1 month after operation, and strenuous exercise was avoided for 3 months. X-ray films of the affected limbs were reviewed every four weeks after discharge to evaluate fracture healing, guide patients in performing knee joint function exercises, record knee joint activity, and record surgical complications. Fracture healing was determined by the X-ray examination results and clinical results. The X-ray examination results showed whether the fracture ends were healed without fracture lines. The clinical results showed whether patients could walk normally without knee pain and therefore whether the fracture was considered to have healed. Bostman scores (Böstman et al., 1981) were used to evaluate knee function at 3- and 9-month follow-ups.

### Statistical analysis

Data analysis was performed using SPSS version 22.0 (SPSS Inc., Chicago, IL, United States). Continuity variables are represented by the mean ± standard deviation. Independent sample *t*-tests were used for comparisons between groups, with *p* < 0.05 considered to be statistically significant.

## Results

### Finite element analysis: Displacement of fractures

**FIGURE 4:**
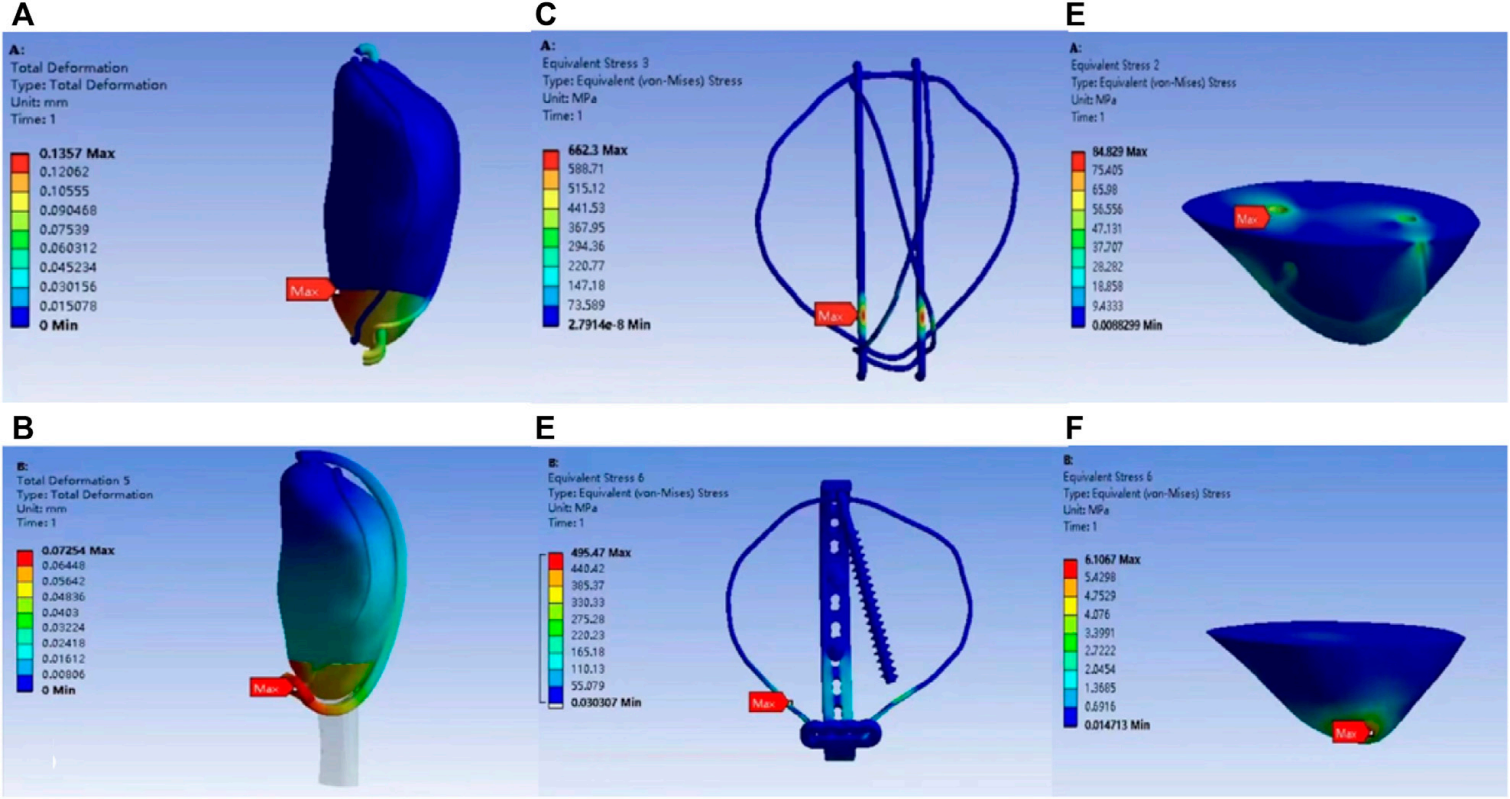
Model displacement distribution program of K-wire group **(A)** and ALP group **(B)**. K-wire group **(C)** and ALP group **(D)** model internal fixation stress distribution program. Stress distribution program of the polar bone block under K-wire group **(E)** and ALP group **(F)** models.

The results of finite element analysis under a 500-N load were as follows. The biomechanical test results of the finite element model showed that the maximum displacements of the K-wire group and the ALP group were 0.136 mm and 0.073 mm, respectively. The maximum displacement of the K-wire group was 1.87 times that of the ALP group, as shown in Figure 4.

### Finite element analysis: Internal fixation and stress distribution of inferior patella pole

The maximum internal-fixation stresses of the K-wire and ALP groups were 662.30 MPa and 495.47 MPa, respectively. The maximum stress of the K-wire group was 1.34 times that of the ALP group. The maximum stresses of the pole bone in the K-wire and ALP groups were 84.83 MPa and 6.10 MPa, respectively. The maximum stress of the pole bone in the K-wire group was 13.91 times that of the ALP group, and the stress was concentrated at each contact point between the tension band and the pole bone. The maximal stress appeared at the point where the K-wire penetrated the pole. The cloud images of the two groups based on finite element analysis are shown in Figure 4.

### Clinical outcome

This study included 49 eligible patients. The average age of the K-wire group was 51.38 years, ranging from 28 to 71 years. The average operation time was 88.48 min, ranging from 72 to 103 min; the average follow-up time was 10.81 months, ranging from 9 to 12 months; the average final knee joint activity was 116.48°, ranging from 102° to 129°; the average Bostman score at 3 months was 22.67, ranging from 21 to 25; and the average Bostman score at nine months was 24.86, ranging from 23 to 26.

The average age of the ALP group was 54.75 years old, ranging from 33 to 75 years old; the average operation time was 75.79 min, ranging from 64 to 89 min; the average follow-up time was 10.46 months, ranging from 9 to 12 months; the average final knee joint activity was 126.75°, ranging from 118° to 135°; the average Bostman score at three months was 26.86, ranging from 24 to 29; and the average Bostman score at nine months was 28.57, ranging from 27 to 30.

**FIGURE 5:**
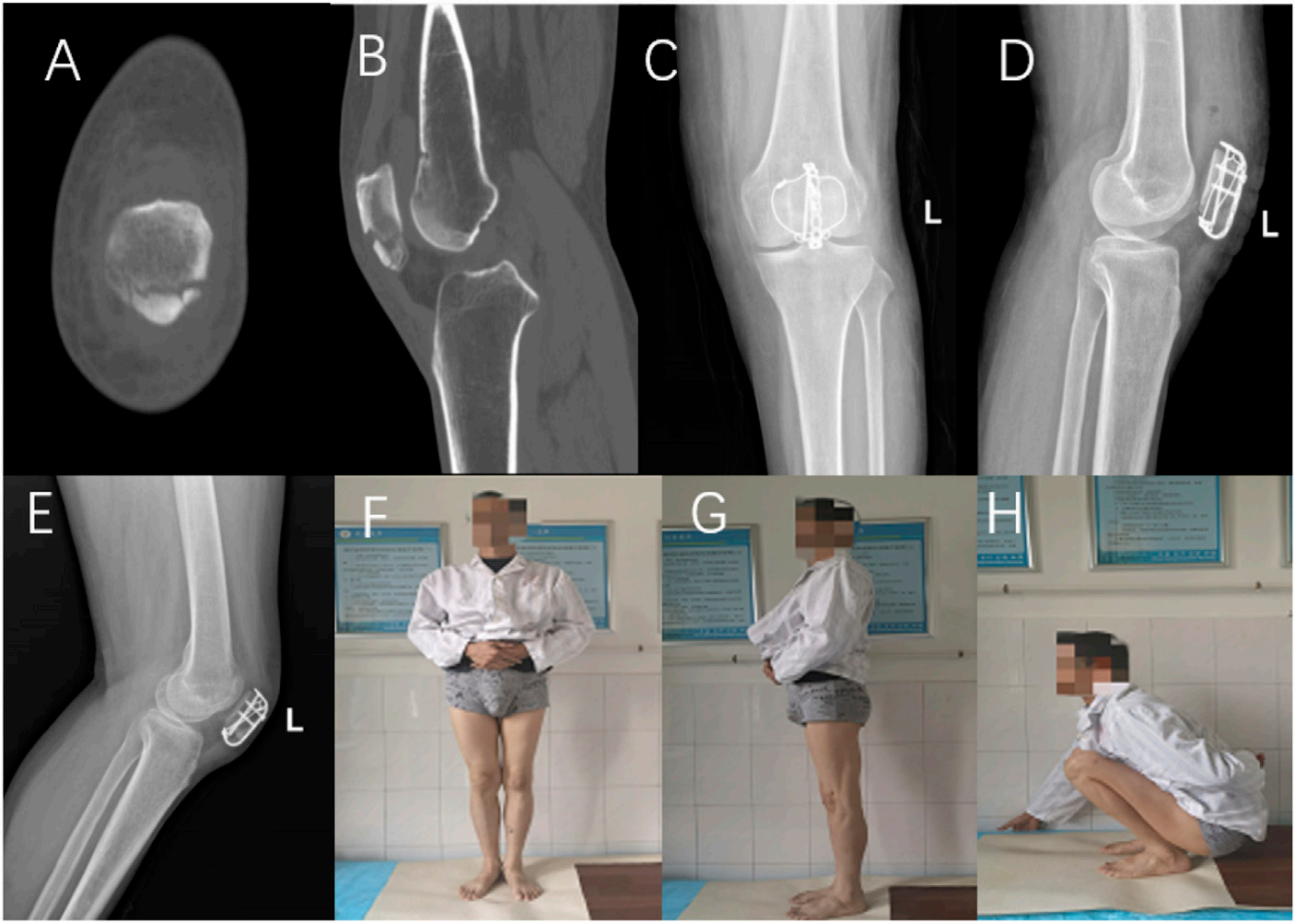
A 56-year-old male presented with a fracture of the inferior pole of the left patella caused by a fall injury. Preoperative CT **(A,B)**, postoperative knee X-ray **(C,D)**. X-ray films and knee joint function 12 months after operation **(E–H)**.

**FIGURE 6:**
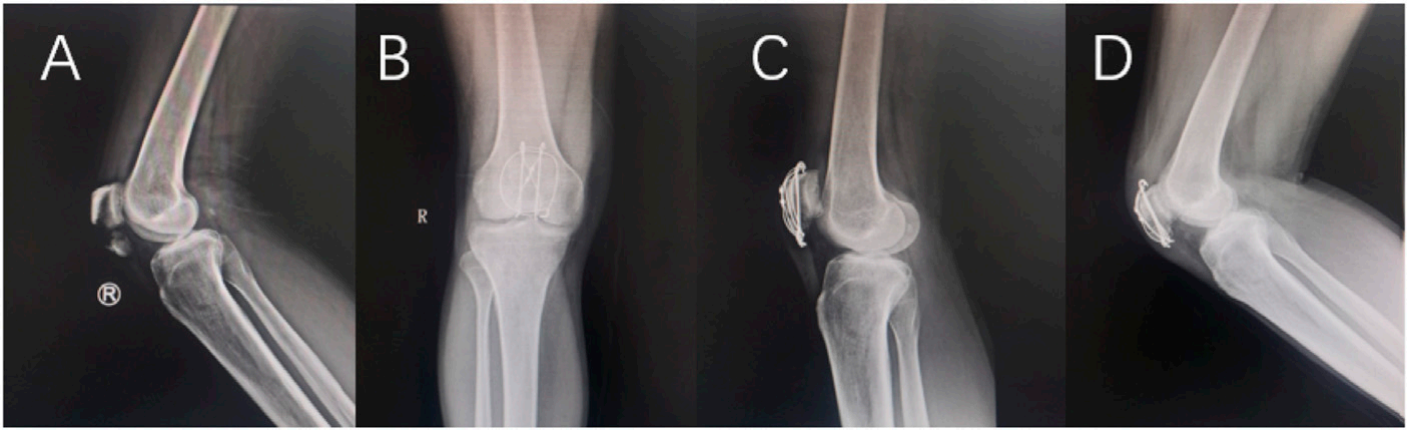
A 45-year-old man suffered a fracture of the inferior pole of the right patella due to a fall. Preoperative X-ray **(A)**, postoperative knee joint X-ray **(B,C)**. The X-ray film **(D)** was reexamined 9 months after operation.

Detailed statistical results are shown in Table 3. Typical cases are shown in Figures 5, 6.

**TABLE 3:** Comparisons of Kirschner-wire tension band combined with patellar cerclage (K-wire) to ALP (ALP) techniques for inferior pole patellar fracture (mean ± SD).

Variables	K-wire (*n* = 21)	ALP (*n* = 28)	*p* Value
Age (years)	51.38 ± 10.31	54.75 ± 10.67	0.273
Follow-up (months)	10.81 ± 1.12	10.46 ± 1.23	0.319
Duration of Surgery(min)	88.48 ± 9.37	75.79 ± 7.17	<0.05
Final ROM	116.48 ± 8.66	126.75 ± 4.87	<0.05
Bostman Score 3	22.67 ± 1.32	26.86 ± 1.35	<0.05
Bostman Score 9	24.86 ± 1.11	28.57 ± 1.07	<0.05

Note: Bostman Score 3 and Bostman Score 9 represent Bostman scores at the third and ninth months of follow-up, respectively.

### Complications

In the ALP group, X-ray examination three months after operation showed bony healing of the lower patella pole and recovery of the range of motion. During the follow-up period, one patient had local soft-tissue stimulation, and their internal-fixation device was removed 1 year after the operation. There were no serious complications such as infection, reduction loss, or internal-fixation displacement. Three patients in the K-wire group had local soft-tissue irritation symptoms, and the internal-fixation device was removed 1 year later. One patient had an incision infection and underwent secondary debridement surgery. The specific complications and secondary surgeries of the two groups are shown in Table 4.

**TABLE 4:** Postoperative complications according to surgical technique.

Technique	*n*	Infection n (%)	Internal fixation stimulation n (%)	Reoperation n (%)
K-wire	21	1 (4.8%)	3(14.3%)	4 (19.0%)
ALP	28	0 (0%)	1 (3.6%)	1 (3.6%)
Total	49	1 (2.0%)	4 (8.1%)	5 (10.2%)

## Discussion

The proportion of infrapatellar fracture among all patellar fractures is not high. However, due to the lack of anatomical structural characteristics in the patellar articular cartilage, these fractures are often crushed, and because the infrapatellar fracture is closely connected to the patellar ligament, the stress is relatively concentrated. Therefore, the treatment of infrapatellar fracture inevitably has some related complications. Until now, in fractures of the inferior pole of the patella, especially in severely comminuted fractures, it has been difficult to fix the fracture fragments of the inferior pole of the patella by internal fixation. For a long time, in cases of extremely severe comminuted fractures under the patella, partial resection of the patella and suture repair of the patellar ligament were preferred due to the lack of suitable fixation methods. However, resection of the inferior pole of the patella is often accompanied by complications such as knee stiffness and patellofemoral arthritis. Veselko and Kastelec (2005) reported that 14 patients with inferior-pole fractures needed plaster fixation for an average of 6.5 weeks after resection of the inferior pole of the patella. Long-term plaster fixation increases the risk of knee stiffness, and the formation of the patellar ligament–bone connection after resection of the inferior pole of the patella is far less than the strength of the bone–bone connection. A retrospective study involving 52 patients found that resection of the inferior pole led to abnormal patellar height and narrowing of the range of activity, which may lead to the problem of patellofemoral arthritis caused by stress concentration of the patellofemoral joint (Bonnaig et al., 2015). There are many complications of partial patellar resection and patellar ligament reconstruction, so the study of internal fixation has become the focus of the treatment of patellar fracture.

Another surgical method is to retain the patellar length in internal-fixation surgery; most surgeons tend to use internal fixation to retain the patellar fracture fragments. The tension band technique is a traditional fixation method for the treatment of patellar fractures. A study by Chang et al. showed that tension-band fixation of patellar inferior-pole fractures showed low nonunion rates and reduced loss rates (Chang et al., 2021), but the sample size was small. Tension-band technology can be applied to the fixation with relatively complete cortical bone support and tensile force (Jang et al., 2019). Although these two conditions are difficult to meet in the tension-band fixation of comminuted patellar fractures, a tension band was applied to the treatment of this fracture, but the clinical effect was poor (Matthews et al., 2017). Bostman et al. found that higher degrees of fracture crushing and older patient ages led to worse surgical effects of the tension band (Böstman et al., 1981). In a study of 52 patients receiving tension bands in the treatment of inferior-pole patellar fracture (Tian et al., 2011), Tian et al. found that eight people had steel wire displacement and three people needed secondary surgery after 1–3 years of follow-up. A study with an average follow-up of 6.5 years found that in the long term, tension-band technology in the fixation of patellar fracture would have internal-fixation failure, nonunion, and other related complications (LeBrun et al., 2012), so tension-band technology was not the best treatment for inferior-pole patellar fracture. However, good surgical results have been achieved through the improvement of tension-band technology.

A more popular improved tension band scheme is to combine a tension band with patellar cerclage (Meng et al., 2021; Wang et al., 2021). Anand et al. (2010) used a suture anchor to fix the patellar fracture. The suture material is an ultra-high molecular weight polyethene fiber with ultra-high knot strength and higher wear-resistance characteristics, and it achieved good therapeutic effects. However, a study by He et al. (2021) showed that it is difficult to achieve strong fixation of all small fractures under sustained stress in the patellar ligament. In determining how to treat inferior-pole patellar fractures, a new method of internal fixation of basket-shaped steel plates provides a new idea. Compared with cases of patellar resection, a follow-up of 4.6 years showed that the basket-shaped plate group was significantly better than the patellar-resection group in knee pain and range of motion (Kastelec and Veselko, 2004). Similar to the method used in this study, the tibial tubercle and proximal patella were fixed directly through the inferior pole of the patella to reduce tension near the fracture end of the inferior pole of the patella; this provides a good healing environment for the fracture. A recent meta-analysis has shown that the fixation effect of steel plates is equivalent to that of tension bands. Compared with steel-plate fixation, the complications and revision rates of tension-band fixation are significantly increased (Raja et al., 2021). Steel plates have achieved good results in the treatment of inferior-pole patellar fracture. The application of a basket-shaped steel plate is one of the most used steel-plate fixation methods for the treatment of inferior-pole patellar fracture, but it is not a perfect scheme. A mechanical study showed that the ultimate load of basket-shaped steel plates is not more than 400 N. When the load is too large, it is easy to cause patellar ligament damage (Krkovic et al., 2007). Because of the limitations of specific internal fixation requirements, it is difficult to widely promote this method in clinical practice. However, the fixed method used in this study has no obvious shortcomings at present.

The patella is an integral part of the knee extension device. The fracture of any part of the patella will undoubtedly damage or completely destroy the function of the knee extension device. Therefore, the purpose of the operation should be to ensure recovery of the continuity of the knee extension device. Most previous surgical fixation methods have aimed to fix the broken ends of the fracture, and the fracture of the lower pole of the patella is often comminuted. When the fracture is fixed with a traditional tension band, if the bone needle penetrates the crushing part of the lower pole, the role of the fixation is minimal. If the patient immediately carries out functional knee joint exercises, bone nonunion, internal fixation failure, and other related complications can easily result. Therefore, it may take a period after surgery to fix the plaster or brace to perform knee joint-function exercise, even if the lower pole is not seriously crushed. The surgical fixation effect is still acceptable. In the early stage of functional exercise, there are also situations such as internal-fixation cutting and nonunion because the stress is concentrated near the fracture line. Jang et al. applied the plate to lower-pole fractures and achieved an excellent therapeutic effect (Jang et al., 2019), finding that the fixation of the plate had higher strength than that of the tension band, which is especially suitable for patellar fractures close to the edge or comminuted. However, its goal is still simply to fix the fracture block.

In this study, the advantages of the ALP were different from the traditional concept of fixing fracture blocks together. The patellar ligament connected to the lower pole is directly connected to the patellar bone bed through the anchor plate, which effects the immediate recovery of the knee extension device. The principle is similar to bridge fixation, which reduces tension around the fracture line so as to provide a good healing environment for the fracture, and forms a temporary and complete knee extension device with an anchor plate as a bridge. This can enable patients to perform early functional exercises to achieve a good recovery effect for knee function in the later period. As early as the beginning of the 20th century, some scholars reported a kind of patellar-tibial external fixation, which directly connected the non-fractured part of the patellar fracture line to the fixation of tibial nodules and achieved the effect of immediate recovery of the knee extension device. After the operation, patients could walk normally under load and the surgery achieved a good therapeutic effect (Petermann et al., 2001). However, its limitation was that the operation was difficult to implement when the patellar fracture was combined with soft tissue swelling around the knee joint and the skin condition was poor. The fixation concept of the method used in this study was similar, and the limitation of incision fixation was small. In the clinical study of plates combined with wire cerclage, we found no internal-fixation failure, inferior pole-bone blockage, bone nonunion, or other related complications in the short term. Biomechanical studies have shown that the maximal load transferred by the knee extension device is about 316 N (Patel et al., 2000), while the finite element analysis in this study showed that the ALP group could receive 500 N prepatellar tension resistance, and the ALP group was more stable than the K-wire group. The stress of the subpatellar bone mass was significantly smaller, which could effectively solve the problem of subpatellar bone-mass cutting. The principles of drag reduction and fixation significantly improved the advantages of the biomechanical properties. Patients in the ALP group had a better prognosis. These results were obtained based on the innovation of internal-fixation devices and surgical methods.

Through the stress analysis of the lower-pole bone in the finite element model of the K-wire group, we found that the contact surface between the tension band combined with patellar cerclage and the lower-pole bone was small, and the stress was concentrated at each contact point between the tension band and the lower-pole bone. The maximum stress occurs in the contact position between the patellar lower pole and the K-wire, where there is a risk of internal fixation failure. The finite element analysis comprehensively compared the biomechanical stability of the two groups, and we found that the displacement of the model, the stress distribution of the internal fixation, and the stress distribution of the lower-pole bone in the ALP group were better than those in the K-wire group. Combined with clinical data from long-term follow-ups, we found that the ALP group had fewer complications, and the surgical effect was satisfactory due to its advantages of mechanical stability, which allowed patients in the ALP group to start early postoperative knee-function recovery. The Bostman scores showed that the patients in the ALP group could obtain good knee joint function in the early stage, resulting in a shorter recovery cycle and higher patient satisfaction.

Our self-made ALP can effectively fix the comminuted lower pole-bone block, especially for severe comminuted lower-pole fractures. We can use additional sutures to suture the lower-pole bone block and the transverse armhole of the anchor plate as a whole and then reset it by traction and hanging. Unlike repeated adjustment, drag reduction can reduce the risk of secondary damage to the original comminuted lower-pole fracture block. Through anchor-plate traction, hanging fixation—not in the lower-pole fracture block into internal fixation, to avoid internal fixation into the lower-pole compression fracture area of bone—results in further iatrogenic fracture, unreliable internal fixation, and other related situations. Ringed steel wire can provide a better lifting and compression fixation effect from both sides and can strengthen the connection between the patella and the internal and external patellar retinaculum. The knee extension device is formed immediately, and its strength is enough to support patients in performing flexion and extension functions earlier. Because of this, patients can attain good knee function after proper rehabilitation training. Finite element analysis shows that the bone plate and steel wire are wrapped in the lower-pole bone from multiple angles, effectively dispersing the stress of the lower pole and avoiding cutting. Theoretically, this can reduce the loss of the lower pole and the probability of internal fixation failure, which is consistent with the results of our clinical observation.

**FIGURE 7:**
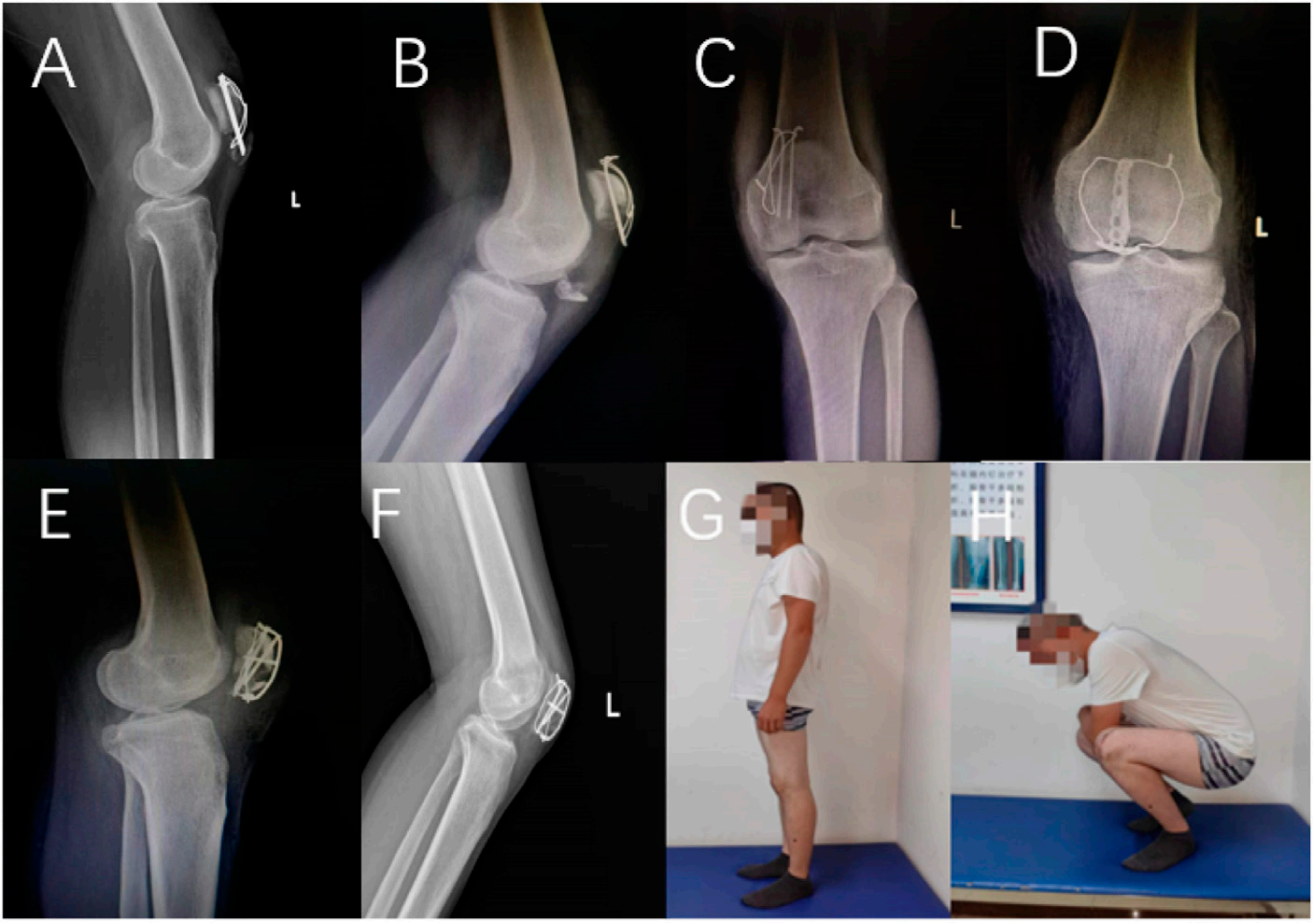
A 66-year-old female patient underwent tension band fixation for two months after the review results **(A)**, and a 29-year-old male patient underwent tension band fixation for one month after the review results **(B,C)**. The above female patient chose partial patellar resection and patellar ligament reconstruction surgery, and the above male patient chose to use the ALP for secondary fixation. Two days after the review results **(D,E)** and three months after the review results **(F)**. At 9 months after operation, the knee joint function had recovered well **(G,H)**.

Most internal fixations in various new treatment schemes for patellar fracture are unique and difficult to popularize. A prominent advantage of our anchor plate is that the internal fixation itself is more popular, but it is only necessary to further shape the internal fixation. Therefore, this new surgical method is easier to popularize. In addition, as shown in Figure 7, we applied the ALP technique to the secondary surgery of the traditional tension band fixation failure and found a good surgical effect. This may provide new possibilities for internal fixation failure after patellar inferior-pole fracture surgery.

In addition, the finite element analysis results given above are based on simulation of the integrity of the lower pole of the patella. For fractures of the lower pole of the patella with severe crushing of the lower pole, the crushing of the loose lower-pole bone weakens the holding force of the tension-band K-wire through the lower-pole bone, making it more likely to cause related complications such as cutting off the lower-pole bone. This increases the difficulty of the operation and decreases the stability of fixation. In our study, seven patients (25%) in the ALP group were aged between 62 and 75 years old, and their postoperative rehabilitation was good. Older patients usually have varying degrees of osteoporosis, and fracture fragments are more likely to be crushed. This study suggests that our surgical plan may be more suitable for elderly patients with patellar fracture and lower pole crushing than is tension band combined with patellar cerclage.

In summary, this method can effectively fix the fracture of the inferior patellar pole, especially in comminuted fractures of the inferior patellar pole with small fracture blocks. Functional exercise can be performed soon after operation to obtain good knee joint function and avoid the occurrence of related complications such as nonunion, internal fixation failure, and inferior patellar pole prolapse. However, the sample size of this study is small, the follow-up time is short, and it is difficult to evaluate long-term efficacy and complications. The stress analysis of the finite element model is only a simple simulation of the stress of the longitudinal axis of the patella, and the real force of the patella involves the articular surface of the patella. The experimental results of the finite element analysis have a certain guiding significance for clinical practice.

## Data Availability

The original contributions presented in the study are included in the article/Supplementary material; further inquiries can be directed to the corresponding author.
